# Eating the Dead to Keep Atherosclerosis at Bay

**DOI:** 10.3389/fcvm.2017.00002

**Published:** 2017-01-30

**Authors:** Megan L. Brophy, Yunzhou Dong, Hao Wu, H. N. Ashiqur Rahman, Kai Song, Hong Chen

**Affiliations:** ^1^Department of Biochemistry and Molecular Biology, University of Oklahoma Health Sciences Center, Oklahoma City, OK, USA; ^2^Karp Family Research Laboratories, Vascular Biology Program, Harvard Medical School, Boston Children’s Hospital, Boston, MA, USA

**Keywords:** atherosclerosis, apoptosis, efferocytosis, macrophages, autophagy

## Abstract

Atherosclerosis is the primary cause of coronary heart disease (CHD), ischemic stroke, and peripheral arterial disease. Despite effective lipid-lowering therapies and prevention programs, atherosclerosis is still the leading cause of mortality in the United States. Moreover, the prevalence of CHD in developing countries worldwide is rapidly increasing at a rate expected to overtake those of cancer and diabetes. Prominent risk factors include the hardening of arteries and high levels of cholesterol, which lead to the initiation and progression of atherosclerosis. However, cell death and efferocytosis are critical components of both atherosclerotic plaque progression and regression, yet, few currently available therapies focus on these processes. Thus, understanding the causes of cell death within the atherosclerotic plaque, the consequences of cell death, and the mechanisms of apoptotic cell clearance may enable the development of new therapies to treat cardiovascular disease. Here, we review how endoplasmic reticulum stress and cholesterol metabolism lead to cell death and inflammation, how dying cells affect plaque progression, and how autophagy and the clearance of dead cells ameliorates the inflammatory environment of the plaque. In addition, we review current research aimed at alleviating these processes and specifically targeting therapeutics to the site of the plaque.

## Introduction

Cardiovascular disease is the leading cause of morbidity and mortality in the United States and its prevalence is rapidly increasing in developing countries (1). Atherosclerosis, the process of vascular wall thickening and hardening, is the primary cause of coronary heart disease (CHD), ischemic stroke, and peripheral aterial disease (2, 3). Patients currently receive therapeutic cocktails containing statins, aspirin, adrenaline β-receptor inhibitors, and angiotensin-converting enzyme inhibitors. However, these patients are still confronted with a 70–80% risk of developing a major acute cardiovascular event (2, 4). Therapies currently available primarily focus on alleviating hypertension and low-density lipoprotein (LDL) cholesterol levels while ignoring the rampant levels of inflammation and other causes of cell death in arterial walls and their consequences on atherosclerotic progression. This inflammation and cell death drives the transition of the stable plaque to a vulnerable plaque, which is prone to rupture leading to thrombosis (5, 6). The instability of the plaque is ultimately a result of oxidized LDL (oxLDL) and its pro-inflammatory effects on endothelial cell activation and macrophage recruitment and function (6). These oxLDLs are sequestered in the subendothelium where maladaptive inflammatory responses to these oxLDLs result in the recruitment, infiltration, and differentiation of monocytes into phagocytic macrophages (7–10). While these macrophages are initially beneficial by clearing the subendotheium of these lipoproteins, the macrophages eventually become engorged with lipids resulting in dysregulated lipid metabolism and a shift in macrophage phenotype to that of lipid-laden foam cells (7, 10, 11). These foam cells eventually undergo apoptosis and necrosis, and, when not cleared efficiently, release their toxic and pro-inflammatory contents into the subendothelial space further promoting cell death and inflammation and further increasing the vulnerability of the plaque (3). Thus, the discovery of new molecules or pathways that inhibit or reduce inflammation, cell death, and dyslipidemia would significantly advance efforts to develop new and more effect therapies to treat this devastating and prevalent disease. Thus, our goal is to analyze several causes of macrophage cell death in the plaque including inflammation, cholesterol metabolism, endoplasmic reticulum (ER) stress, and mechanisms of opposing the inflammatory environment of the plaque and clearing dead cells, namely efferocytosis and autophagy in addition to emerging therapies aimed at these processes.

Atherosclerosis most often develops in areas of low shear stress with disturbed or oscillating flow, typically areas with branch points, bifurcations, or curvatures (12, 13). In these areas of disturbed flow, endothelial cells develop a pro-inflammatory phenotype; they exhibit increased production of reactive oxygen species, increased cell turnover and permeability, shorter glycocalyx, and increased apoptotosis. Furthermore, they exhibit increased NF-κB signaling, increased expression of leukocyte adhesion molecules, increased production of fibronectin, and increased secretion of MCP-1 (13). These endothelial cells are further activated by an environment of hyperlipidemia. LDLs in the circulation interact with proteoglycans in the extracellular matrix of the endothelial cells where they become sequestered and undergo modifications such as aggregation and oxidation becoming proatherogenic (14). Exposure of endothelial cells to oxLDL induces the expression of MCP-1 (CCL2) and VCAM-1 (6).

**Figure 1:**
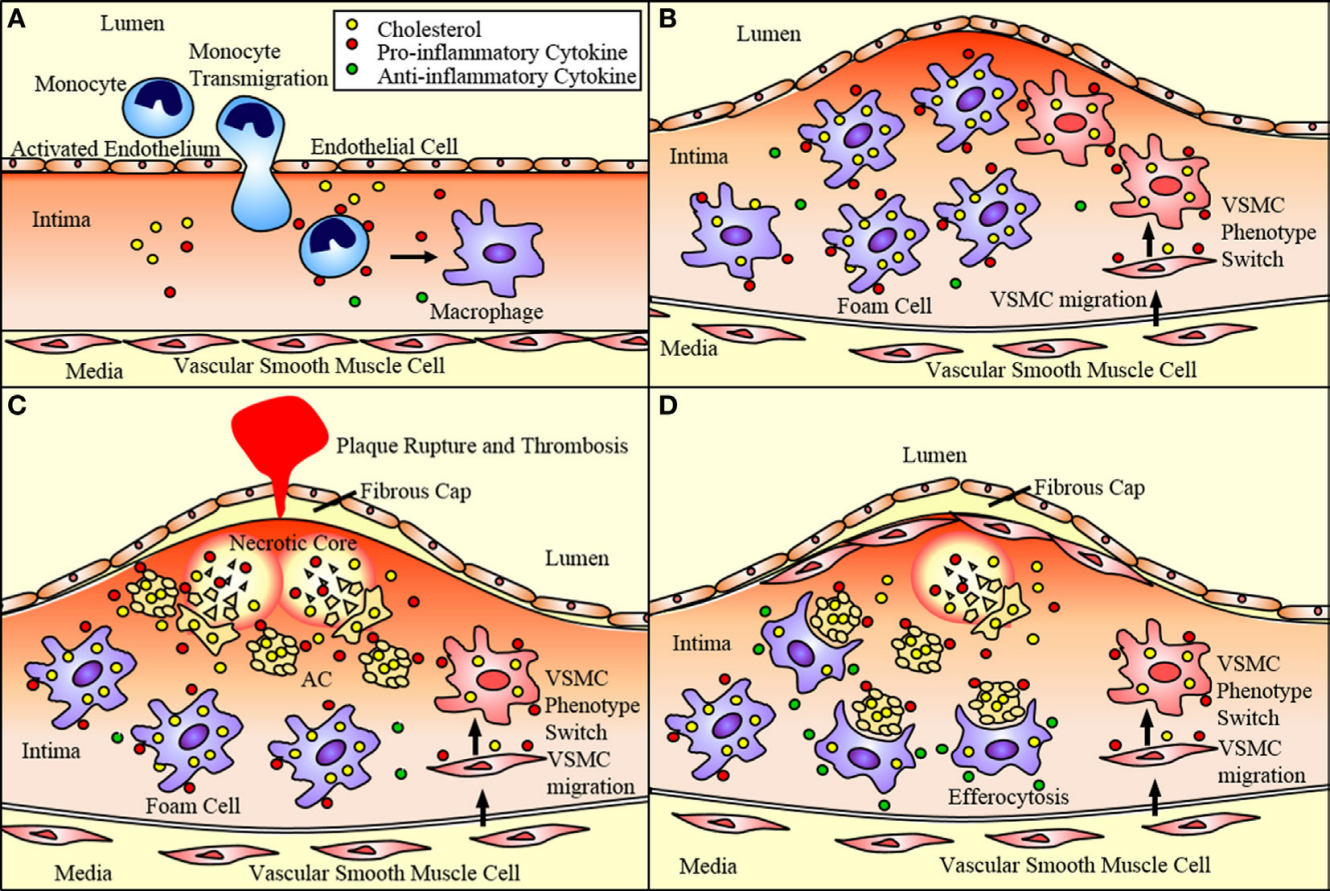
**Atherosclerotic plaque progression**. **(A)** Endothelial cells activated by disturbed flow and oxidized low-density lipoprotein (oxLDL) uptake express adhesion molecules and chemoattractant proteins that recruit monocytes and promote their adhesion to the endothelium and transmigration into the subendothelium. These monocytes differentiate into macrophages. **(B)** Macrophages uptake oxLDL sequestered in the subendothelium eventually becoming lipid-laden foam cells. Vascular smooth muscle cells (VSMCs) can migrate to the subendothelium where they lose expression of SMC markers and gain expression of macrophage markers. This allows them to ingest lipids and eventually become foam cells contributing to plaque progression. **(C)** These foam cells eventually undergo apoptosis and necroptosis, and, if not effectively cleared by M2 macrophages *via* efferocytosis, undergo secondary necrosis contributing to the formation of the necrotic core. As the necrotic core grows and the fibrous cap thins, the plaque is vulnerable to rupture, which may result in acute cardiovascular events such as thrombosis. **(D)** VSMCs near the cap of the plaque secrete extracellular matrix components that contribute to the formation of a fibrous cap that protects the plaque from rupturing. M2 macrophages express anti-inflammatory markers that act to reduce the inflammation of the plaque. They also perform efferocytosis, thereby reducing the apoptotic and necrotic cells within the plaque and promoting plaque stability.

P- and E-selectin expressed by endothelial cells loosely bind circulating monocytes causing them to role along the endothelium as they break and reform bonds with the receptors PSGL-1 and ESL-1 (15, 16). Chemokines sequestered in the endothelial glycocalyx activate integrins strengthening the adhesion of the monocytes and promoting their transmigration from the lumen of the vessel to the subendothelium (15). MCP-1 is one such chemokine that aids in the recruitment of monocytes expressing CCR2, the MCP-1 receptor (16). Once in the subendothelium, the monocytes differentiate into phagocytic macrophages (Figure 1A), which are classically activated to pro-inflammatory M1 macrophages or alternatively activated to anti-inflammatory M2 macrophages. These macrophages ingest the lipids sequestered in the subendothelium becoming engorged and eventually leading to dysregulated lipid metabolism and foam cell formation (7, 10, 11). These foam cells secrete pro-inflammatory cytokines including MCP-1, which recruit additional monocytes and macrophages in a positive feedback mechanism (15, 16). These foam cells accumulate within the subendothelium giving rise to the plaque (Figure 1B). As these foam cells undergo apoptosis or necroptosis, a necrotic core forms within the plaque (Figure 1C). Plaques with a thin fibrous cap and large necrotic cores are likely to rupture leading to thrombosis, heart attack, or stroke (5, 17). Furthermore, macrophages play a primary role in the clearance of dead and dying cells within the plaque enabling plaque regression (Figure 1D). Thus, macrophages play a critical role in the progression of atherosclerotic plaques. Particularly, the balance between macrophage death and the clearance of dead cells by macrophages is a determining factor in plaque progression and vulnerability.

Additionally, vascular smooth muscle cells (VSMCs) play an ever expanding role in the pathogenesis of atherosclerosis. VSMCs traditionally produce collagen forming the fibrous cap of the lesion and preventing plaque rupture and thrombosis (18) (Figure 1D). However, pro-inflammatory cytokines released by activated endothelial cells and macrophages promote the alteration of the VSMC phenotype to that of a migratory macrophage-like phenotype. These macrophage-like VSMCs engulf lipids becoming foam cells and contributing to plaque progression (16) (Figure 1).

## Macrophage Death in the Atherosclerotic Plaque

In the atherosclerotic lesion, activated macrophages contribute to the progression of the lesion by secreting inflammatory cytokines (19). In advanced lesions, dead and dying macrophages are not efficiently cleared leading to secondary necrosis in which the cells become leaky and swollen eventually releasing their contents into the subendothelium. This process eventually results in the formation of the necrotic or lipid core, which is itself an inflammatory stimuli to other macrophages in the subendothelium further eliciting inflammatory responses and causing cellular damage (20). Along with the necrotic core, these activated macrophages also promote the thinning of the fibrous cap by releasing pro-inflammatory cytokines, ROS, and matrix metalloproteinases (21). This may eventually lead to the rupture of the fibrous cap exposing tissue factor to its ligand and resulting in acute cardiovascular events (17, 22, 23).

Several mechanisms are responsible for cell death within the plaque. These include both intrinsic and extrinsic apoptosis and necroptosis. Apoptosis is driven by the activation of caspases, which lead to cell rounding, chromatin condensation, nuclear membrane fragmentation, a reduction in cellular volume, and membrane blebbing (24). The extrinsic pathway is triggered by extracellular signals including damage associated molecular patterns and cytokines. These triggers are sensed by death receptors, toll-like receptors, and NOD-like receptors, which propagate apoptosis signaling *via* caspase 8 (24, 25). The intrinsic pathway is triggered by intracellular signals such as ER stress, oxidative damage, and DNA damage among others. This pathway propagates apoptotic signaling *via* caspase 3 and caspase 7 (24). Necroptosis is regulated necrosis, which leads to cellular swelling and plasma membrane rupture. This pathway is activated in response to death receptors, genotoxic stress, and viruses and signals through RIPK1, RIPK3, and MLKL (24, 26).

Cell death occurs in lesions throughout the progression of atherosclerosis. Gautier et al. demonstrated that apoptotosis in early lesions is atheroprotective but promotes inflammation and further plaque progression in advanced lesions. This study used ApoE-deficient mice overexpressing Bcl2, a protein which inhibits the intrinsic apoptotic pathway. At 5 weeks on Western diet, these mice exhibited large lesions with increased numbers of macrophages compared to control mice. However, at 15 weeks on Western diet, the plaques were smaller with decreased numbers of macrophages (27). The extrinsic apoptotic pathway also plays a significant role in atherosclerosis. The Fas ligand has been shown to be enriched in regions of the plaque that are TUNEL positive or apoptotic (28). Furthermore, this pathway has recently been linked to ER stress-induced apoptosis specifically in macrophages. ER stress induces the release of calcium from the ER. These high cytosolic levels of calcium cause the activation of CAMKII, which can then induce apoptosis *via* Fas signaling (29). In addition, treating mice with a necroptosis inhibitor greatly reduces plaque size and instability (30).

In early lesions, the dying cells are most likely and effectively cleared by neighboring macrophages performing efferocytosis, the phagocytosis of dying cells, resulting in smaller lesions with fewer macrophages. However, in advanced lesions, the vast majority of dying cells in need of clearance are macrophages (31–34). Since macrophages are the primary cell type responsible for efferocytosis within the plaque, it is likely that the large lesion size and necrotic core result from inefficient clearance of the dying cells and macrophage death itself, likely caused by cholesterol loading and ER stress.

## Efferocytosis in Macrophages

**Figure 2:**
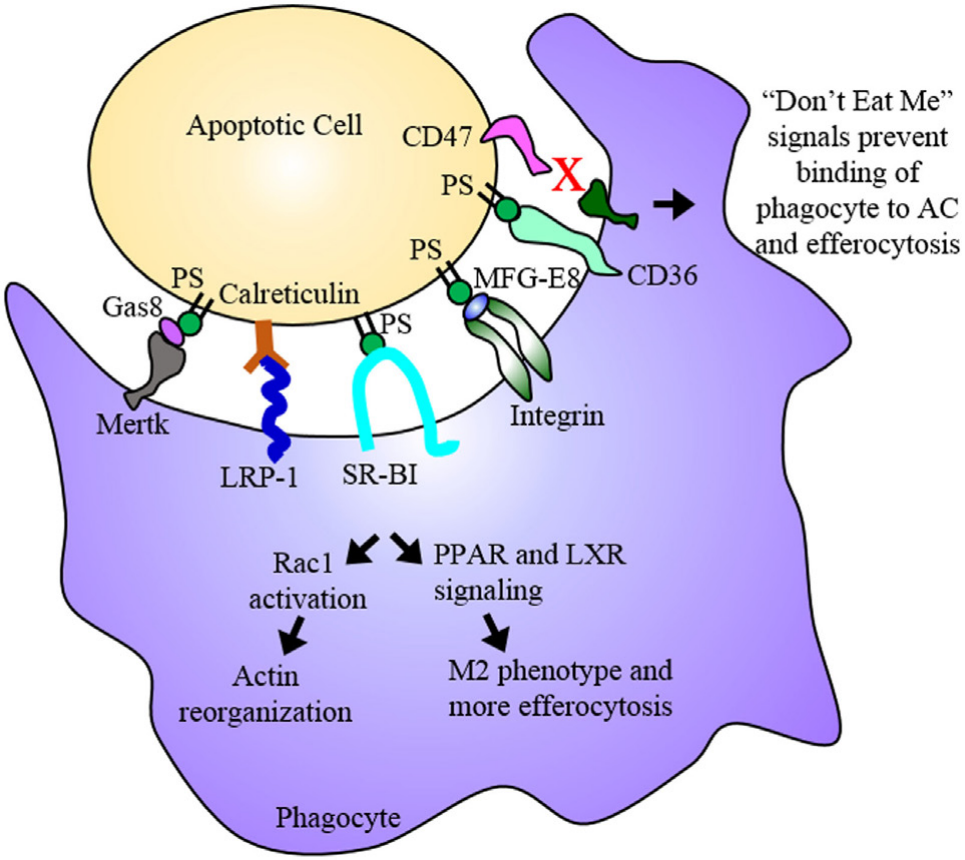
**Efferocytosis**. Efferocytosis is the phagocytosis of dying cells by macrophages and is an essential process for the maintenance or regression of the atherosclerotic plaque. Phosphatidylserine (PS) among other molecules are “eat me” signals expressed on the dying cell, which are recognized by receptors on the phagocyte. Binding of PS by these receptors results in Rac1 activation and actin reorganization as well as PPAR and LXR signaling. PPAR and LXR signaling result in the upregulation of proteins involved in binding the dying cell as well as proteins involved in cholesterol efflux and anti-inflammatory cytokines ultimately promoting an anti-inflammatory M2 macrophage phenotype. The dying cell is engulfed and digested, its inflammatory contents cleared from the subendothelium. In advanced plaques, apoptosis and efferocytosis become dysregulated and imbalanced. Apoptotic cells that express “don’t eat me” signals evade uptake by phagocytes and eventually undergo secondary necrosis contributing to the development of the necrotic core.

Efferocytosis is the clearance of dead and dying cells by phagocytes. Efferocytosis functions to clear cells in early stages of cell death while the plasma membrane is still intact. It also prevents secondary necrosis, thereby preventing the extracellular release of the cytotoxic and inflammatory contents of the dying cell (35). Dying cells release “find me” signals such as fractalkine or CXC3CL1, which establish a chemotactic gradient that stimulates the phagocyte to migrate toward the dying cell (36, 37). The dying cells also display “eat me” signals on their surface, which are recognized by specific receptors on the phagocyte. These ligand receptor pairs include calreticulin or complement C1q with LRP-1, thrombosopondin and CD36, and phosphatidylserine (PS) with SR-BI, integrins, and TIM-4 among others (37). Once the phagocytic receptor binds its ligand on the dying cell, a series of signaling events occur in the phagocyte that result in the activation of Rac-1, actin rearrangement, phagocytic cup formation, and engulfment of the dying cell (Figure 2). Once the dying cell is internalized, it and its contents are degraded (38). There are two main methods of engulfment: the phagocytic membrane is extended around the dying cell and closes like a zipper forming a tight fitting phagosome (39, 40), while necrotic cells are internalized in a fluid-filled phagosome much like macropinocytosis (40, 41). However, both methods converge on the same signaling pathways for phagocyte recognition and actin rearrangement (42). Thus, efferocytosis, in the context of the atherosclerotic plaque, acts to reduce the apoptotic and necrotic burden within the plaque, thereby reducing the probability of the formation of a vulnerable, rupture prone plaque. Furthermore, efferocytosis actively promotes an anti-inflammatory environment within the plaque. Treatment of primary macrophages with fractalkine prior to co-incubation with apoptotic thymocytes induced expression of MFG-E8, a bridging molecule involved in the binding of the phagocyte to the apoptotic cell, and increased phagocytosis (43). Through the ingestion of dying foam cells, efferocytosis provides phagocytic macrophages with large amounts of lipids, which are PPAR and LXR ligands (44). PPAR and LXR signaling induce the expression of genes involved in sensing “eat me” signals such as C1q, a bridging molecule, and Mertk, an efferocytic receptor (45). This increase in Mertk further stimulates an increase in its corresponding bridging molecule, Gas6 (45, 46). LXR signaling *via* apoptotic cell recognition also induced the expression of ABCA1 and ABCG1 resulting in a significant increase in cholesterol efflux from macrophages (16, 19, 47, 48). PPAR and LXR signaling also result in the increased production and release of anti-inflammatory signals such as IL-10, induces anti-inflammatory M2 macrophage polarization, and suppresses pro-inflammatory signals such as TNFα and IL-1β as well as inhibiting NFκB gene transcription, which is a key mechanism of M1 activation (49–53). Thus, efferocytic signaling further enhances efferocytosis and acts to resolve the inflammation present within the plaque (Figure 2).

Recent studies have made it apparent that, in the advanced plaque, efferocytosis is dysregulated, either through the overabundance of dying cells or the inability of macrophages to efficiently perform phagocytosis. Severe deficiency in efferocytosis has been demonstrated in human atherosclerotic plaques (54). Despite the numerous receptors and ligands involved in apoptotic cell clearance and their apparent overlap in function, simply disrupting one receptor can cause a significant deficiency in efferocytosis. Several studies have demonstrated that reduction of LRP-1, specifically in macrophages, or its ligand, calreticulin, significantly reduces efferocytosis while simultaneously enhancing the inflammatory phenotype of macrophages, foam cell formation, and plaque progression in mice (55–57). Another study used LDLR null mice, which were treated with anti-TIM-1 and anti-TIM4 antibodies to block TIM-1 and TIM-4 binding to PS and thereby inhibiting efferocytosis. These mice exhibited increased atherosclerotic lesion size as well as increased macrophage content within lesions. TIM-4 blockade specifically impaired efferocytosis as demonstrated in cultured primary peritoneal macrophages, while TIM-1 blockade promoted proatherogenic Th1 cells (58). Furthermore, transplanting SR-BI null myeloid cells into ApoE null mice fed Western diet significantly increased macrophage apoptosis within the lesion as well as lesion necrosis. This also resulted in reductions of efferocytosis and Rac-1 signaling (59). While it is known that oxLDL reduces the expression of SR-BI, the exact mechanisms regulating the expression of proteins involved in efferocytosis are not entirely clear (60). Thus, illuminating mechanisms that downregulate or upregulate proteins involved in efferocytosis is critical for the development of new therapies. Molecules present in the pro-inflammatory environment have been shown to regulate efferocytosis. Mertk, has been shown to undergo shedding, or release of its extracellular domain, when exposed to metalloproteinases, which are highly expressed in the lesion. Mertk shedding resulted in reduced apoptotic cell clearance (61). When ADAM17, the primary metalloproteinase responsible for Mertk shedding, is genetically deleted from mice, primary macrophages exhibit an M2 anti-inflammatory phenotype with increased efferocytosis and increased resolution of inflammation (62). These studies demonstrate that the pro-inflammatory environment within the plaque can impair efferocytosis by downregulating the expression or function of proteins involved in the clearance of dead and dying cells. Conversely, a recent study by Kojima et al. demonstrated that blocking CD47 reduces plaque burden and enhances efferocytosis in mice. CD47 functions as an antiphagocytic “don’t eat me” signal that is upregulated in atherosclerosis (41). When ApoE null mice fed Western diet were treated with anti-CD47 antibodies to block CD47 function, these mice exhibited decreased lesion size, decreased necrotic core area and caspase activity indicative of decreased cell death, and increased plaque stability. Furthermore, *in vitro* efferocytosis analysis with primary macrophages and staining of aortic root lesions demonstrated increased efferocytosis. The authors went on to demonstrate that the expression of CD47 is induced by TNFα, a pro-inflammatory cytokine present within the plaque. In addition, treatment of primary VSMCs with TNFα reduced their susceptibility to apoptosis as well as reducing their efferocytic uptake by primary macrophages (63). Developing molecules to downregulate other such “don’t eat me” signals presents another potential therapeutic strategy.

## Cholesterol Metabolism in Macrophages

Reverse cholesterol transport is the transport of cholesterol back to the liver from the circulation for excretion in the bile and feces. Macrophages play an essential role in the process both through cholesterol uptake and cholesterol efflux. The receptors primarily responsible for the uptake of oxLDL are CD36, SR-AI, SR-AII, and LOX-1. OxLDLs are also taken up by micropinocytosis, which is mediated by TLR4. After the lipoproteins are transported into the macrophage, the cholesteryl esters are hydrolyzed to free cholesterol in late endosomes. The free cholesterol is then esterified by ACAT1 and stored in the ER as lipid droplets. The cholesterol esters undergo secondary hydrolysis prior to being transferred out of the cell *via* passive diffusion or scavenger receptors ABCA1, ABCG1, and SR-BI during cholesterol efflux (64, 65).

In atherosclerosis, the process becomes dysregulated. In conditions of hyperlipidemia, activated macrophages uptake large amounts of oxLDL. However, this does not downregulate the expression of CD36, the SR-As, or LOX-1. In fact, several pro-inflammatory cytokines prominent in the plaque including IFN-γ, TNF-α, and IL-1β, promote the upregulation of the SR-As, CD36, and LOX-1 while downregulating ABCA1 and ABCG1 (66), thereby enhancing cholesterol uptake while simultaneously inhibiting cholesterol efflux and contributing to enhanced foam cell formation. When these foam cells accumulate an excess of free cholesterol, they can undergo apoptosis or necrosis contributing to plaque progression, necrotic core formation, and plaque rupture (67–69). Furthermore, macrophages engulf cholesterol crystals, which form in the arterial wall. These crystals can trigger inflammasome activation *via* NLRP3 leading to IL-1β secretion (70). This further perpetuates the cycle of inflammation and macrophage death within the plaque ultimately leading to plaque progression. In addition, when macrophages undergo apoptosis due to free cholesterol accumulation, they induce the production of the pro-inflammatory cytokines TNF-α and IL-1β in competent phagocytic macrophages. This results in a pro-inflammatory M1 phenotype (71). Thus, the dysregulation of cholesterol metabolism in macrophages acts to promote the pro-inflammatory environment within the plaque as well as macrophage death resulting in plaque progression and the formation of the necrotic core.

## ER Stress in Endothelial Cells and Macrophages

Under normal conditions, IRE1, PERK, and ATF6, three transmembrane proteins, extend through the ER membrane into the ER lumen. In the lumen, they are inactive when bound to chaperones involved in protein folding (16). Ischemia, hypoxia, excess protein synthesis, ROS, and excess free cholesterol, and disordered lipid composition within a cell can lead to ER stress (72, 73). In response to ER stress, protein chaperones are upregulated and PERK and IRE1 become oligomerized and activated initiating the unfolded protein response (UPR) (74, 75). The UPR acts to reduce protein synthesis, enhance protein degradation, and increase protein folding ultimately resulting in the reduction of stress in the ER. Specifically, PERK acts to suppress general protein translation, IRE1 activates inflammatory and survival signaling pathways, and ATF6 is processed by proteases in the Golgi apparatus forming a transcription factor, which upregulates ER chaperones (74–76). However, continued ER stress and UPR activation can lead to cell death.

Under normal conditions, endothelial cells create barrier separating molecules circulating in the blood stream from the subendothelium. However, as the endothelial cells become activated, this endothelial barrier begins to break down becoming leaky. In addition to the transendocytosis of lipoproteins into the subendothelium, this further enables circulating lipoproteins to be taken up into the subendothelium and modified (77). This endothelial cell dysfunction also results in the production of chemokines that recruit monocytes to the subendothelium and contribute to an environment that enables the phenotypic switching of VSMCs resulting in their increased proliferation and increased production of extracellular matrix components, which contribute to the fibrous cap of the plaque (78). ER stress has been shown to be a prominent cause of endothelial cell dysfunction. Disturbed flow, known to activate the endothelium, may also promote ER stress in endothelial cells. Phospho-ATF6 and phospho-IRE1 have been shown to be upregulated in endothelial cells in regions of disturbed flow (79); the phosphorylation of these two proteins is indicative of ER stress and activation of the UPR. Furthermore, endothelial cells exposed to disturbed flow have a larger burden of ROS compared to endothelial cells not exposed to disturbed flow; ROS is known to induce ER stress in endothelial cells (79), OxLDL and disturbed flow in endothelial cells has been shown to upregulate the expression of LOX-1, the primary receptor in endothelial cells responsible for the uptake of oxLDL (80, 81). OxLDL has also been demonstrated to activate endothelial cell CHOP and caspase 12, both involved in the activation of the UPR (82). Thus, disturbed flow and oxLDL activate the endothelium possibly by inducing ER stress in endothelial cells eventually leading to endothelial cell dysfunction.

Several studies have demonstrated the contribution of ER stress and the UPR to macrophage death and necrotic core formation within atherosclerotic plaques. At all stages of lesion development, the UPR is activated. Markers for UPR activation, including phospho-PERK and CHOP, along with free cholesterol accumulation have been observed in both early and advanced atherosclerotic lesion of mice (83). Furthermore, exposure of THP-1 macrophages to oxLDL induced intracellular accumulation of 7-ketocholesterol, which upregulates ER chaperones and CHOP expression leading to UPR activation. Furthermore, this treatment caused the macrophages to undergo oxidative stress and increase the expression and secretion of MMP9 (84). Oxidative stress promotes cellular damage contributing to the inflammatory environment of the plaque and cell death while MMP9 degrades the extracellular matrix weakening the fibrous cap and creating a rupture prone plaque. In addition, cholesterol trafficking to ER membranes has been shown to induce the UPR and the activation of CHOP, which is induced by ER stress and mediates apoptosis. This occurs through the depletion of ER calcium stores, which has also been demonstrated to activate the UPR. Furthermore, CHOP null macrophages were protected from cholesterol-induced death (72). Thus, in hyperlipidemia, oxLDL uptake and cholesterol loading of macrophages in the plaque initially clears the subendothelium of these pro-inflammatory stimuli but eventually results in the dysregulation of cholesterol metabolism, ER stress, cell death, necrotic core formation, and plaque rupture. The clearance of these macrophages is of the upmost importance in preventing the progression of the plaque and eventual acute cardiovascular events.

## Autophagy in Endothelial Cells, VSMCs, and Macrophages

Under non-pathological conditions, autophagy functions to degrade large cellular structures and aggregated proteins. Autophagy consists of two steps beginning with the formation of the autophagosome, a double membrane vesicle that engulfs cytoplasm containing misfolded proteins or damaged organelles (85, 86). These autophagosomes shed their coat proteins and then fuse with lysosomes forming a single membrane autolysosome. The contents are degraded and recycled for anabolic reactions within the cell (85–87). When functioning appropriately, autophagy reduces apoptosis within the plaque, thereby increasing plaque stability (85, 86). However, several studies have shown that dysregulation of autophagy in multiple cell types is associated with atherosclerosis.

Properly functioning autophagy is essential for lipid homeostasis in endothelial cells. Knockdown of Atg7, a protein involved in autophagosome elongation, in HUVECs resulted in the accumulation of intracellular oxLDL. Endothelial cell-specific knockout of Atg7 in ApoE null mice also demonstrated intracellular accumulation of oxLDL with increased atherosclerotic lesion size containing larger necrotic areas (88). Several studies have demonstrated that disturbed flow and oxLDL not only promote endothelial cell activation but dysregulation of autophagy. Significant downregulation of Beclin1, LC3II, and LC3I were observed in endothelial cells exposed to disturbed flow compared to those exposed to physiological shear stress (89). Given that Beclin-1 is required for autophagosome formation and the LC3 proteins coat the autophagosome aiding in lysosome fusion, endothelial cell exposure to oxLDL results in the dysregulation of autophagy, thereby enhancing the pro-inflammatory environment of the plaque (85). HUVECs treated with oxLDL exhibit damaged mitochondrial DNA (mtDNA). Under pro-atherosclerotic conditions when autophagy is defective, this mtDNA leaks into the cytoplasm and initiates an inflammatory response dependent on TLR9 (90). Furthermore, VSMCs exposed to low levels of oxLDL exhibited increased expression of LOX-1 and increased autophagy. However, when the concentration of oxLDL was increased to reflect the loss of cellular defenses in atherosclerotic plaques, autophagy and apoptosis were induced (91). 7-Ketocholesterol is found in atherosclerotic plaques and is known to induce ER stress. When VSMCs are treated with 7-ketocholesterol, they accumulate ubiquitinated proteins, thereby activating the UPR and eventually leading to defective autophagy and cell death (92). Thus, autophagy is an essential process for maintaining lipid homeostasis but one that is susceptible to dysregulation in an atherosclerotic environment.

Atg5 is another protein involved in the elongation of the autophagosome (87). When this protein is selectively knocked out in macrophages in ApoE null mice, macrophages exhibit increased apoptosis and oxidative stress but decreased phagocytic clearance *in vitro* and promote plaque necrosis, macrophage apoptosis, and oxidative stress within the plaque (93). Another study using this mouse model demonstrated that advanced plaques exhibit markers consistent with dysfunctional autophagy. In addition, macrophage-specific deficiency of Atg5 increased the cholesterol crystal burden within the plaque as well as the activation of the macrophage inflammasome further promoting the progression of atherosclerosis in these mice (94). Furthermore, decreased autophagy has been demonstrated in foam cells. Treatment of macrophages with the autophagy activator rapamycin exhibited reduced lipid content resulting in reduced foam cell formation *in vitro* (95). Thus, the dysregulation of autophagy may be a contributing factor to the formation of lipid-laden foam cells in the progression of atherosclerosis. Agents that enhance the function of authophagy may prove vital for the improvement of treatments for patients with CHD.

Lipophagy is a form of selective autophagy in which lipid droplets are delivered to the lysosome for degradation and has been demonstrated in hepatocytes (96). It is now understood that this process also occurs in macrophages and has an impact on cholesterol metabolism. It has been previously shown that foam cells deliver a portion of their neutral lipids to lysosomes for hydrolysis in addition to the cytoplasm (97). Furthermore, lipid droplets sequestered in autophagosomes have been demonstrated to be delivered to lysosomes. Here, the lipid droplets undergo hydrolysis *via* lysosomal acid lipase to form free cholesterol, which is then effluxed out of the cell (98). Agents that can induce this pathway of cholesterol metabolism may enhance cholesterol efflux, thereby reducing foam cell formation and plaque progression.

## VSMC Phenotype Switching and Contribution to Plaque Progression

Historically, the role of VSMCs in atherosclerosis has been simple: their proliferation promotes plaque formation and their production of collagen, which expands the extracellular matrix of the endothelium and forms the fibrous cap (99). However, recent studies suggest a far more complex role for these cells in the progression of atherosclerosis. Several studies have shown that cultured VSMCs could gain a macrophage-like phenotype while losing expression of characteristic VSMC markers as evidenced by co-staining of smooth muscle actin (SMA) and CD68 (macrophage marker) in human atherosclerotic lesions (100–103). However, these studies were unable to determine if these cells were of smooth muscle origin or not. A genetic inducible fate-mapping study using ApoE null mice in which smooth muscle cells and their progeny exhibited β-galactosidase activity used both Western blot and tissue staining to demonstrate the upregulation of macrophage markers MAC-2 and CD68 and downregulation of smooth muscle cell markers SMA and smooth muscle myosin heavy chain with concomitant β-galactosidase activity in cultured VSMCs derived from atherosclerotic aortas compared to non-atherosclerotic aortas (104). A second smooth muscle cell lineage tracing study showed that over 80% of VSMCs, approximately 30% of total cells, within atherosclerotic lesions are undetectable using conventional SMA staining. These cells undergo a phenotypic transition within the lesion to a more macrophage-like phenotype. Furthermore, the loss of KLF4 within SMCs resulted in a reduction of VSMCs with a macrophage-like phenotype within the lesion in addition to reduced lesion size and reduced fibrous cap thickness. This study also elucidated a role for VSMCs in foam cell formation. Upon cholesterol loading, KLF4 expression and expression of macrophage markers were induced in VSMCs resulting in phagocytic behavior and the expression of pro-inflammatory cytokines (105). Much like macrophages, these phenotypically altered VSMCs exhibit expression of SR-As, CD36, and LOX-1 and downregulation of ABCA1 and ABCG1 indicating increased oxLDL uptake and reduced cholesterol efflux resulting in foam cell formation (106–108). Furthermore, oxLDL has been shown to activate the TLR4-mediated inflammatory signaling pathway in VSMCs. This results in the upregulation of ACAT1 expression further promoting intracellular lipid accumulation and foam cell formation (109). These lipid-laden VSMCs can also undergo cholesterol-induced cell death resulting in the release of MCP-1, fractalkine, and other pro-inflammatory cytokines that recruit monocytes, promoting further VSMC phenotype switching, and enhancing plaque development (110). Given the similarities between these phenotypically altered VSMCs and macrophages, it is probable that the same mechanisms lead to cell death, namely ER stress, and dysregulation of cholesterol metabolism and autophagy. Furthermore, VSMCs likely play a significant role in plaque progression by contributing to the foam cell formation, cell death, and necrotic core formation within the plaque.

## Therapeutic Implications and Future Directions

Many current therapies used to treat patients with cardiovascular disease focus on ameliorating hypertension and lowering lipid levels. However, these treatments yield less than ideal results. New treatments that address the high levels of inflammation and cell death and low levels of dead cell clearance may help to fill this gap. Much research has been done to examine the effectiveness of reducing ROS and inflammation in atherosclerosis as therapeutic options for CVD. While it is enticing to develop therapies to reduce cell death within the atherosclerotic lesion, this may ultimately prove detrimental. As previously stated, apoptosis in early lesions reduces plaque size while apoptosis in more advanced lesions promotes the formation of the necrotic core and increases plaque vulnerability. Furthermore, the digestion of these apoptotic cells by phagocytic macrophages promotes anti-inflammatory signaling and cholesterol efflux, two processes which promote plaque regression. Considering that eliminating one efferocytic receptor on macrophages significantly impairs efferocytosis as a whole, developing therapies that enhance efferocytosis, either by promoting “eat me” signals or their receptors, may be a more effective therapeutic strategy.

ROS contribute both to inflammatory signaling in the atherosclerotic plaque and ER stress in endothelial cells, macrophages, and VSMCs. Thus, antioxidant therapies designed to counter ROS may prove effective in reducing plaque progression, inflammation, and cell death. Several studies have focused on compounds found in food that have been shown to have antioxidant properties such as vitamins C and E. Vitamin C scavenges ROS, reduces pro-inflammatory cytokine levels, and prevents endothelial cell apoptosis (111). Several forms of vitamin E have been shown to reduce ROS production by inhibiting NADPH oxidase and scavenge-free radicals (112). Various studies in humans with vitamins C and E have shown conflicting results showing beneficial, neutral, and harmful effects of these compounds, which are extensively reviewed elsewhere (113–115). However, the contrasting results among these studies could be due to differences in doses, time course, patient populations, the use of food-derived versus synthetic vitamins, and the use of different forms of Vitamin E, not all of which have the same effects (114, 116). Thus, further studies with more standardized methods or utilizing more targeted delivery methods may be needed to truly evaluate the potential of vitamins C and E as therapies for atherosclerosis.

Several other therapies have been developed recently to combat the role of ROS in the development and progression of atherosclerosis. Xanthine oxidoreductase (XOR) is highly expressed in atherosclerotic plaques and is known to produce ROS and promote the progression of atherosclerosis (117–119). When atherosclerotic mice are treated with the XOR inhibitor febuxostat, they exhibit reduced atherosclerotic lesion size and macrophage infiltration into the plaque, and reduced ROS levels in the aortic wall, as well as reduced expression of pro-inflammatory genes within the aorta (120). Thus, inhibiting the enzymes responsible for ROS production may prove to be a valuable therapeutic option for CVD. Tian et al. synthesized quercetin 7-0-sialic acid to combine the ROS scavenger sialic acid and quercetin, which has been shown to be an anti-inflammatory antioxidant that promotes cholesterol efflux. This compound protected HUVECs from hydrogen peroxide and oxLDL-induced oxidative damage by reducing the production of ROS. It also reduced the expression of the adhesion molecules ICAM1 and VCAM1 as well as the pro-inflammatory cytokines TNFα and MCP-1 in these cells, suggesting that aortic endothelial cells treated with this agent would recruit fewer monocytes. Furthermore, RAW264.7 cells, a mouse macrophage-like cell line, exhibited upregulation of ABCA1 and ABCG1 resulting in increased cholesterol efflux when treated with 7-0-sialic acid (121). While both quercetin and sialic acid have anti-atherosclerotic properties on their own, combining these into one compound greatly increased their effects (121). Thus, combining other molecules with individual antiatherosclerotic properties may prove an effective method for creating new, more effective treatments for CVD. Szeto and Schiller developed SS-31, a peptide that acts as an antioxidant by scavenging reactive oxygen species specifically in the inner mitochondrial membrane (122). This peptide is currently in phase II clinical development; however, its potential use in treating cardiovascular disease has only recently been demonstrated (123). SS-31 has been shown to both reduce oxLDL accumulation, at least partially by downregulating CD36 and LOX-1, and suppress oxLDL-induced ER stress in RAW264.7 cells. Furthermore, treatment of RAW264.7 cells with this peptide reduced oxLDL-induced inflammation by reducing IL-6 and TNFα secretion (124). While both of these compounds are promising, further study, particularly whole animal studies, need to be completed to provide a clearer picture of their effectiveness in treating CVD.

Enhancement or inhibition of several molecules found in mammals have also shown promises as potential therapies for CVD. Growth differentiation factor 11 (GDF11) is associated with lower risk of CVD, but its levels decline in circulation with age (125). When this growth factor was administered to ApoE null mice on high fat diet, endothelial cells from these mice exhibited reduced endothelial dysfunction and reduced apoptosis of primary endothelial cells. GDF11 also reduced atherosclerotic lesion size in mice as well as pro-inflammatory cytokine expression in RAW264.7 cells (126). Thus, GDF11 represents a promising potential therapy to ameliorate multiple aspects of atherosclerotic plaque progression. Pro-resolving mediators, such as Resolvin D1, have been shown to inhibit pro-inflammatory signaling and enhance anti-inflammatory signaling (127). Resolvin D1 has been shown to be significantly reduced in both human vulnerable plaques and advanced murine plaques most likely due a mechanism involving oxidative stress. When atherosclerotic mice are treated with Resolvin D1, oxidative stress and necrotic core size within the plaque are reduced while cap thickness and efferocytosis are increased (128). Promoting the resolution of inflammation is a promising therapeutic area to affect multiple aspects of plaque progression. TREM-1 is a receptor expressed on monocytes and macrophages, which has been shown to enhance the production of pro-inflammatory cytokines and chemokines such as CCL2 and TNFα, which are involved in the promotion of plaque progression, while hindering the release of anti-inflammatory cytokines such as IL-10 (129). A recent study found that atherosclerotic mice with a TREM-1 deficiency exhibited reduced macrophage recruitment, necrotic core size within plaques, reduced foam cell formation, reduced pro-inflammatory cytokine secretion, and ultimately reduced plaque size compared to control mice. Furthermore, these results were replicated by treating atherosclerotic mice with the LR12 peptide, which inhibits TREM-1 binding to its endogenous ligand, these mice (130). Thus, blocking the agents of inflammation, namely monocytes, from entering the atherosclerotic plaques represents a viable and potent therapy for atherosclerosis.

A significant challenge in CVD therapy development is treating cells in areas affected in the disease or specific cell types as opposed to multiple cell types throughout the body. Furthermore, targeting treatments, such as antioxidants and resolving mediators, to specific cell types, organelles, or specific regions of the body may enhance their effectiveness in ameliorating atherosclerosis. Recent work has focused on this issue of targeting therapies and has shown promising results with molecules targeted to the mitochondria aimed at resolving oxidative stress originating from this organelle. MitoQ, a derivative of the antioxidant ubiquinone conjugated to a lipophilic cation, enters and accumulates within the mitochondria (131–133). This molecule has been shown to reduce endothelial dysfunction and reduce not only cellular and mitochondrial ROS but also the secretion of pro-inflammatory cytokines (134, 135). Furthermore, when atherosclerotic mice that were also haploinsufficient for ATM were treated with MitoQ, they exhibited decreased fat accumulation and oxidative stress, and improved lipid and glucose metabolism compared to control treated mice. Despite a lack of change in atherosclerotic plaque size, macrophage content, apoptosis, and oxidative damage were significantly reduced within the plaques at least indicating a more stable plaque compared to control treated mice (136). Mitochondrial-targeted Vitamin E has been shown to reduce oxidative stress and apoptosis is primary endothelial cells (137). Furthermore, several studies in mouse models of sepsis have shown this molecule to reduce oxidative stress and inflammatory cytokine production (138, 139). Esculetin is another antioxidant whose functions have gained increased efficacy through mitochondrial targeting. Karnewar et al. demonstrated that this compound alleviated oxidative stress and cell death by increasing the phosphorylation of eNOS resulting in increased NO production in human aortic endothelial cells. Furthermore, atherosclerotic mice treated with this molecule exhibited reduced plaque size and reduced monocyte and macrophage infiltration into the plaque (140). While studies involving mitochondrial-targeted antioxidants are promising, more studied are needed both in atherosclerotic and human models. Resolving mediators, such as annexin A1, function to terminate inflammation and promote tissue repair (141). Fredman et al. recently designed an amino terminal peptide that mimics the function of annexin A1. Nanoparticles were used to deliver this peptide specifically to sites of injury using a collagen IV-binding peptide. When LDLR null mice were treated with this compound, the nanoparticles were detected in atherosclerotic lesions within aortic root sections with reduced detection at other sites such as the spleen and liver. This amino terminal peptide resulted in increased fibrous cap thickness and suppression of oxidative stress within the plaque as well as reduced necrosis. Ultimately, the site-specific delivery of this peptide resulted in reduced atherosclerotic lesion size in these mice (142). Chung et al. identified a novel peptide that binds specifically to endothelial cells exposed to disturbed flow. To evaluate this peptide as a potential therapeutic strategy, it was used to deliver siRNA targeting ICAM-1 in a partial carotid ligation mouse model. This resulted in reduced ICAM-1 expression in aortas under conditions of disturbed flow potentially resulting in reduced monocyte recruitment (143). Thus, nanoparticles and targeted peptides greatly enhance the ability to specifically treat atherosclerotic prone regions and represent a rapidly growing area in cardiovascular research.

## Conclusion

Atherosclerosis is, in part, a progressive inflammatory disease that is exhibited by increased apoptosis and defective clearance of dead cells. Both cholesterol metabolism and ER stress contribute to these processes. Given the complexity of atherosclerosis progression, it is not surprising that current treatments are largely ineffective. Current therapies ignore the inflammation and cell death within the atherosclerotic lesion. Unfortunately, these processes drive plaque progression and vulnerability. Thus, elucidating the pathways that control this feedforward cycle of inflammation and death may illuminate new molecules that can aid in the development of new therapies. Such pathways worthy of focus are cholesterol metabolism, cytokine production, and ER stress all of which promote a pro-inflammatory environment and cell death. Furthermore, enhancing the proper recycling of damaged organelles and lipids *via* autophagy as well as augmenting efferocytosis either through the downregulation of “don’t eat me” signals or the upregulation of “eat me signals” and their receptors may also prove to be effective therapeutic options.

## Author Contributions

MB wrote the manuscript and created the figures. YD, HW, HR, KS, and HC contributed to discussions and preparing the manuscript.

## Conflict of Interest Statement

The authors declare that the manuscript was constructed in the absence of any commercial or financial relationships that could be construed as a potential conflict of interest.
